# HSP27 modulates tumoural immune evasion by enhancing STAT3-mediated upregulation of PD-L1 and NLRC5 in ovarian cancer

**DOI:** 10.3332/ecancer.2023.1526

**Published:** 2023-03-31

**Authors:** Maha Fezza, George Hilal, Roula Tahtouh, Malak Moubarak, David Atallah

**Affiliations:** 1 Cancer and Metabolism Laboratory, Faculty of Medicine, Saint-Joseph University, B.P. 17-5208 - Mar Mikhael, Beirut 1104 2020, Lebanon; 2 Department of Gynecology and Obstetrics, Hôtel-Dieu de France University Hospital, Achrafieh Area, Beirut 166830, Lebanon; 3 Department of Gynecology and Gynecologic Oncology, Evangelische Kliniken Essen Mitte, Henricistraße 92, Essen 45136, Germany

**Keywords:** ovarian cancer, PD-L1, NLRC5, HSP27, STAT3

## Abstract

Multiple preclinical studies have demonstrated that the addition of hyperthermia (HT) to immunotherapy could enhance tumour immunogenicity and stimulate an antitumour immune response, primarily via heat shock proteins (HSPs). However, antitumour immune responses are often impeded by immune evasion mechanisms, such as the overexpression of programmed death-ligand1 (PD-L1) and the loss of major histocompatibility complex class 1 (MHC-1) expression. In this context, we sought to investigate the effect of HT on PD-L1 and NOD-like receptor family CARD domain containing 5 (NLRC5) identified as the key transcriptional activator of MHC-1 genes, and their interaction in ovarian cancer. A coculture of ovarian cancer cell lines (IGROV1 and SKOV3) with peripheral blood mononuclear cells was set up. Then, culture media conditioned with IGROV1 or SKOV3 subjected to HT was tested on untreated cell cultures. Knocking down heat shock protein B1 (HSPB1 or HSP27), heat shock protein A1 (HSPA1 or HSP70), and pharmacological inhibition of STAT3 phosphorylation were performed. Subsequently, we measured expression levels of PD-L1, NLRC5, and proinflammatory cytokines. The correlation between PD-L1 and NLRC5 expression in ovarian cancer was evaluated using the Cancer Genome Atlas database. We found that HT produces a concomitant decrease in PD-L1 and NLRC5 expression in coculture. Notably, however, the conditioned media by heat-shocked cells increases their expression. HSP27 knockdown can reverse this increase. Adding STAT3 phosphorylation inhibitor significantly enhanced the expression inhibition of PD-L1 and NLRC5 induced by HSP27 silencing. Correlation analysis showed a positive correlation in ovarian cancer between NLRC5 and PD-L1. These findings demonstrate that HSP27 modulates PD-L1 and NLRC5 expression through the activation of a common regulator ‘STAT3’. Moreover, the positive correlation between PD-L1 and NLRC5 led us to conclude that the upregulation of PD-L1 and the downregulation of MHC class I are two mutually exclusive mechanisms of immune evasion in ovarian cancer.

## Background

Ovarian cancer is the fifth leading cause of cancer-related- deaths among women after lung, breast, colorectal, and pancreatic cancer [1]. Although its incidence rate has slowly decreased over the years, the mortality rate has remained high [2]. To date, cytoreductive surgery followed by aggressive chemotherapy is considered the gold standard treatment for patients with advanced ovarian cancer [3]. This approach provides the most prolonged survival for patients with no macroscopic disease left after the surgery. However, most patients will ultimately experience disease recurrence [4].

Significant progress has been made recently, and several treatment alternatives have been proposed, such as hyperthermic intraperitoneal chemotherapy (HIPEC) [5]. Combining hyperthermia (HT) with chemotherapy has proven its efficacy in many clinical trials in terms of increased overall survival and decreased recurrence rate in patients with advanced ovarian cancer [6, 7]. The effectiveness of this combination can be mainly attributed to the pleiotropic effects of HT [8]. Enhancing chemotherapeutics delivery through inducing changes in the cytoskeleton, increasing microvascular permeability and tumour perfusion, decreasing tumour cell density and adhesion, and impairing DNA repair have all been reviewed as mechanisms of heat effects [9].

Interestingly, HT also holds great potential for improving immunotherapy outcomes. Increasing evidence has shown that HT is capable of modulating immune responses. In this regard, Lee *et al* [10] reported that heating tumour tissues above body temperature (up to 43°C) regulates innate and adaptive immune systems by inducing immune cell activation and altering cytokines and chemokines expression. Indeed, previous studies have demonstrated that local HT combined with intratumoural injection of dendritic cells (DC) enhances DC migration to tumour-draining lymph nodes and improves T-lymphocytes priming in patients with advanced melanoma [11, 12]. Natural killer cells (NK) have also been reported to be responsive to HT. Ostberg *et al* [13] showed that HT enhances the cytolytic activity of NK cells by inducing the NKG2D clustering, which may strengthen its interaction with its ligand MHC class I polypeptide-related sequence A (MICA), located on the tumour cell surface**.** Moreover, heat can increase blood perfusion to tumours and upregulates the expression of intercellular adhesion molecule-1 (ICAM-1), thus facilitating the infiltration of immune effector cells into tumours [14]. These diverse mechanisms of action unfold many potential strategies combining HT with immunotherapy.

Heat shock proteins (HSPs) are considered to be the primary molecular actors involved in HT immunogenicity. HSPs are classified into several groups based on their molecular size; small HSP (<40 kDa), HSP40, HSP60, HSP70, HSP90, and HSP100-110; among which HSP70 is recognised as immunostimulatory, while HSP27 is recognised as immunodepressive [15]. High amounts of HSPs are generated rapidly in response to environmental stressors [16]. HSPs act as molecular chaperones to promote refolding of stress-damaged proteins, thus allowing the recovery of cellular functions [17]. Apart from their chaperoning activities, HSPs have been reported to inhibit apoptosis pathways in cancer cells and induce resistance to potentially cytotoxic HT via the generation of refractory thermotolerant cells [18]. Also, HSPs were shown to drive angiogenesis by stabilising Hypoxia-inducible factor-1, while some reports indicate that extracellular HSP27 can bind to receptors, directly stimulating the transcription of the Vascular endothelial growth factor [19]. HSPs are then partly responsible for tumour cell survival.

Most importantly, HSPs are increasingly recognised as essential immune response modulators and are considered emerging targets for cancer immunotherapy [20]. When released from stressed cells, HSPs act simultaneously as carriers for antigenic peptides and as danger signals for DC activation [21]. Furthermore, once endocytosed by DC, HSPs stimulate the expression of major histocompatibility complex (MHC) class II and co-stimulatory molecules and increase the release of modulatory molecules, including cytokines IL-12, TNF-α, IL-1β, GM-CSF, thus enhancing immune recognition of antigens [22]. In this regard, Das *et al* [20] reported that the internalisation of the HSP-peptide complex is more efficient than the internalisation of unchaperoned free soluble antigens. In fact, by binding to CD91, which is expressed on most antigen-presenting cells, the HSP-peptide complex initiates a signalling cascade, leading to the production of cytokines and upregulation of co-stimulatory molecules. Considering all of the above, HT seems to affect multiple phases of the cancer immunity cycle, thereby strengthening the antitumour immune response.

However, the role that HT and HSPs play in the regulation of drivers of cancer immune evasion is not yet clear. One key mechanism of immune evasion in ovarian cancer is upregulating programmed death ligand-1 (PD-L1) expression to create an immunosuppressive microenvironment. Around 10%–33% of epithelial ovarian cancer tumours express PD-L1 [23]. Such high expression is correlated with poor prognosis [24]. Indeed, by binding to Programmed death-1 (PD-1) on tumour-infiltrating T-cells (TILs), PD-L1 inhibits signalling pathways downstream of the T-cell receptor (TCR), thus hindering T-cell proliferation, cytolytic function, and cytokines release [25].

Loss of MHC class 1 expression is another common cancer immune evasion strategy that leads to reduced immune recognition by CD8+ T cells. NOD-like receptor family CARD domain containing 5 (NLRC5) has been recognised as the main MHC class I transactivator [26]. Ludigs *et al* [27] demonstrated the selectivity of NLRC5 for genes encoding MHC class 1 molecules and proteins functioning in the MHC class 1 mediated antigen presentation pathway. Impaired function and expression of NLRC5 have been reported in many cancer types, among which ovarian cancer carries the highest frequency of copy number alterations of the NLRC5 gene. Those alterations can impair CD8+ T cells’ cytotoxic activity and lead to a poor prognosis by reducing NLRC5 expression levels [28]. Moreover, Lv *et al* [29] recently demonstrated that NLRC5 expression could predict the response to immunotherapy in melanoma patients. This is consistent with the NLRC5 function of inducing MHC class 1 genes, whose downregulation presents a hallmark of resistance to immunotherapy [30].

Therefore, it is particularly compelling to realise how HT affects these two significant targets of cancer immune evasion mechanisms (PD-L1 and NLRC5) when considering the integration of HT into immunotherapy strategies for treating ovarian cancer.

## Methods

### Cell lines

The human ovarian cancer cell line SKOV3 (HTB-77^™^) was purchased from the American Type Culture Collection (ATCC, Manassas, VA, USA), and maintained in Dulbecco’s Modified Eagle Medium (DMEM)/F12 while being supplemented with 10% fetal bovine serum (FBS), 1% penicillin/streptomycin. The IGROV1 cell line purchased from Sigma-Aldrich (St. Louis, MO, USA) was maintained in DMEM high glucose with 10% FBS and 1% penicillin/streptomycin. All cells were cultured in a humidified atmosphere at 37°C with 5% CO_2_.

### Determination of cell viability

Cells were seeded in 96 well-plates with 100 μL medium and incubated overnight before hyperthermic exposure. The next day, the medium was refreshed and cells were exposed to 42°C or 43°C for 30 minutes, or for 1 hour in a 5% CO_2_ incubator. Cells maintained at 37°C were set as control. At the end of exposure, cells were returned back to 37°C for recovery. Cell viability levels were determined at 4, 24, 48, and 72 hours post exposure, by using the WST-8 cell counting kit; according to the manufacturer’s protocol (Sigma-Aldrich, Germany). This assay uses tetrazolium salt which is converted to a fluorescent product formazan by metabolically active cells. Fluorescence was monitored at 450 nm by ELISA reader Multiskan Go. Cell viability was expressed as a percentage of control cells. Five replicates (*n* = 5) of each experimental condition were performed.

### Exposure to HT

In the subsequent experiments, cells were exposed to 42°C for 1 hour in a standard 5% CO_2_ incubator. Briefly, the appropriate number of cells was plated and incubated at 37°C overnight, while on the next day, the medium was changed prior to hyperthermic exposure as plates were transferred into a 5% CO_2_ incubator set at 42°C and exposed for 1 hour. Then, the plates were returned to a 37°C incubator for recovery during various periods (2, 4, 6, 24 and 48 hours).

### Isolation of peripheral blood mononuclear cells (PBMC)

Blood was collected from five healthy volunteers, who have expressed their informed consent in written form prior to sample collection. The collected blood was placed in K3/EDTA vacuum tubes. Shortly after collection, it was diluted with twice the volume of phosphate-buffered saline (PBS), then layered on an equal volume of Histopaque^®^-1077 (Sigma-Aldrich), followed by centrifugation at 400 g for 30 minutes at room temperature with centrifuge acceleration and break set at its lowest setting. The mononuclear cell layer was carefully transferred to a new tube after aspiration of the upper layer and later subjected to a series of PBS washes. PBMC viability and count were assessed by trypan blue staining. PBMC were suspended in RPMI medium supplemented with 10% FBS and 1% penicillin-streptomyc at a density of 3 × 10^6^ cells/mL.

### Coculture of PBMC with ovarian cancer cells

Corning^®^ Transwell^®^ polyester membrane cell culture inserts (Corning, NY) were used to mimic the *in vivo* state and the tumour microenvironment. Initially, fresh medium was added to the multiple-well plate and the transwell insert; then incubated overnight to enhance cell attachment. Ovarian cancer cell lines (IGROV1 and SKOV3) were then seeded in the transwell insert compartment at the density of 3 × 10^5^ then returned at 37°C in an incubator with humidified atmosphere containing 5% CO_2_. The next day, ovarian cancer cell lines were exposed to 42°C for 1 hour, then PBMC were added to the lower compartment immediately following density gradient isolation at 1/10 ratio of IGROV1 or SKOV3 to PBMC. PBMC and ovarian cancer cells were harvested 24 and 48 hours after hyperthermic exposure (42°C for 1 hour).

### Treatment with conditioned media by heat-shocked cells

Upon reaching 80% of confluence, ovarian cancer cell lines were transferred to a 42°C incubator for 1 hour. After 24 hours of recovery at 37°C, the conditioned media from the heat-shocked cells was collected and subjected to centrifugation at 1,500 rpm for 10 minutes to pellet cellular debris. The conditioned media from non-heat shocked cells was also collected and used as control. Conditioned supernatants were then transferred to ovarian cancer cell lines, which were subsequently cultured for 24 hours. Conditioned media experiments were used to test whether the observed effect under hyperthermic exposure is dependent on a soluble factor secreted by cancer cells.

### Analysis of NLRC5 and PD-L1 expression in patients with ovarian cancer

The correlation analysis between NLRC5 and PD-L1 expression in ovarian cancer was conducted using the GEPIA tool (http://gepia.cancer-pku.cn/) based on The Cancer Genome Atlas (TCGA) database.

### Transient siRNA transfection

First, IGROV1 and SKOV3 cells were seeded at a density of 3 × 10^5^ in 6-well culture plates and incubated under normal growth conditions (37°C and 5% CO_2_). Meanwhile, HSPA1A and heat shock protein B1 (HSPB1) siRNA (QIAGEN) stock solutions (20 µM) were diluted into 100 µL of serum-free medium and 12 µL of Hiperfect transfection reagent (QIAGEN, Germany) mixed and then incubated at room temperature for 10 minutes to allow the formation of transfection complexes. The complexes were added dropwise onto the cells so that the final concentration is 30 nM. AllStars Negative Control (QIAGEN) was used as a negative control, then cells with transfection mix were incubated at 37°C for 48 hours. Thereafter, cells were washed with PBS and incubated with a medium containing 10% serum at 42°C for 1 hour. Knockdown was confirmed by real-time polymerase chain reaction (PCR).

### Cell treatment with STAT inhibitor

Ovarian cancer cell lines were cultured in 96-well plates at a density of 1 × 10^5^ cells per mL. At 80% confluence rate, cells were treated with increasing doses of STAT3 activation inhibitor STATTIC (Tocris Bioscience) 5, 10, and 20 µM or vehicle [dimethyl sulfoxide (DMSO)] for different time periods (4, 24, and 48 hours). The WST-8 assay was performed to determine the optimal concentration for subsequent treatments.

### Cell lysis and western blotting

Protein samples were prepared by lysing cells with radioimmunoprecipitation assay (RIPA) buffer (Abcam) in the presence of sodium orthovanadate, protease inhibitor cocktail, and phenylmethylsulfonyl fluoride (PMSF), all purchased from Sigma. Protein concentration in the supernatants was done by ultracentrifugation using the Microcon^®^ Centrifugal Filter Devices (Merck Millipore). Protein concentration was determined with DC protein assay (Bio-Rad). Equal amounts of protein were separated with 10% Mini-PROTEAN TGX Gel (Bio-Rad) and then transferred to polyvinylidene difluoride (PVDF) membrane (Bio-Rad). The membrane was incubated with HSP27, HSP70, HSP60, PD-L1, STAT3, phospho-STAT3 (Tyr705), beta- actin (Cell Signaling Technology) antibodies at 1:1,000 and NLRC5 rat mAb (Clone3H8, 1:1,000; Merck) overnight. Primary antibodies were detected with anti-rabbit immunoglobulin G (IgG), horseradish peroxidase (HRP)-linked antibody (Cell Signaling Technology) except for the NLRC5 rat monoclonal antibody which was detected with anti-rat IgG, HRP-linked antibody (Cell Signaling Technology) and developed with SignalFire^™^ Enhanced Chemiluminescence (ECL) Reagent (Cell Signaling Technology).

### ELISA detection of IL-1β, IL-12, TNF-α and TGF-β

An enzyme-linked immunosorbent assay was performed to detect and quantify inflammatory cytokines in supernatants from cocultures of ovarian cancer cell lines and PBMC. ELISA kits were purchased from R&D Systems (Human IL-1β/IL-1F2 DuoSet, Human TGF-β DuoSet, Human TNF-α DuoSet, Human IL-12 p70 DuoSet ELISA) and were used according to the manufacturer’s protocol. The optical density of the colorimetric reaction was determined using an ELISA plate reader at 450 nm. Recombinant human IL-1β, IL-12, TNF-α, and TGF-β were used to establish the standard curve for each assay.

### RNA extraction and real-time PCR

Total RNA was extracted using the NucleoSpin^®^ RNA extraction kit (Macherey-Nagel, USA). RNA quality and yields were analysed using a NanoDrop spectrophotometer (ND-1000). iScript™ Reverse Transcription Supermix for RT-qPCR from Bio-Rad was used to synthesise the first-strand cDNA.

Quantification of gene expression was performed by real-time PCR with iTaq Universal SYBR Green Supermix (Bio-Rad) and the primers listed below (Table S1). cDNA was used to measure the expression levels of the following panel of genes: HSP27, HSP70, HSP60, HSP90, IL-1ẞ, IL-12, TGF-ẞ, TNF-α, c-Myc, AKT, STAT3, PD-L1, NLRC5. Primers were designed using the Primer3 tool and the Primer Blast tool was used to check their specificity. The relative expression of these genes was normalised to that of β-Actin. Briefly, 10 μL of the final reaction volume consisting of 1 μL of cDNA at a 5 ng/μL concentration, 5 μL of SYBR green, 3 μL of nuclease-free water, and 0.5 μL of each primer was added to the CFX Connect Real-Time PCR Detection System (Bio-Rad). The cycling conditions were predenaturation at 95°C for 5 minutes, followed by 40 cycles of denaturation at 95°C for 6 seconds and annealing and extension at 60°C for 30 seconds. Melting curves were established at the end of the PCR to assess product purity. The relative expression was calculated using the value of 2^−ΔΔCt^ (Delta Ct = Ct gene – Ct endogenous control, Delta Delta Ct = ΔCt sample1 − ΔCt calibrator).

### Statistical analysis

One-way ANOVA followed by Turkey’s multiple comparisons test were performed to analyse data from biological replicates using GraphPad Prism software. All experiments were repeated independently at least three times. A *p-*value of ˂0.05 was considered statistically significant.

## Results

### Exposure to 42°C for 1 hour had a mild impact on the viability and induced HSP expression which allowed cell recovery

**Figure S1.**
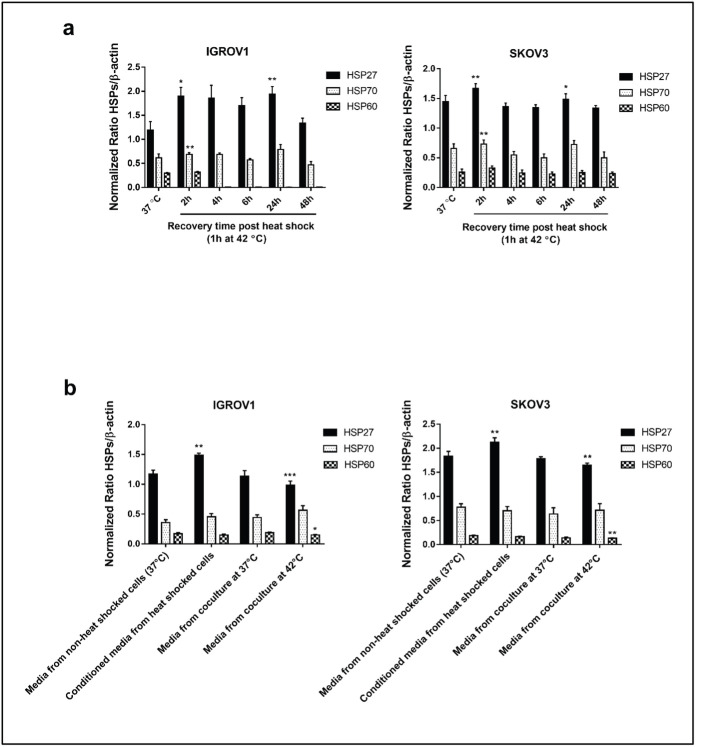
Densitometric analysis of HSPs (HSP27, HSP70 and HSP60) protein levels normalised to β-actin. (a): From the Western blot of supernatants derived from heat-treated cells (42°C for 1 hour) at different recovery time points (Figure 1i). (b): from the Western blot of HSPs in the conditioned media and media from coculture system (Figure 3e). Data are presented as mean ± SD from three independent experiments. *p < 0.05, **p < 0.01, ***p < 0.001.

**Figure 1.**
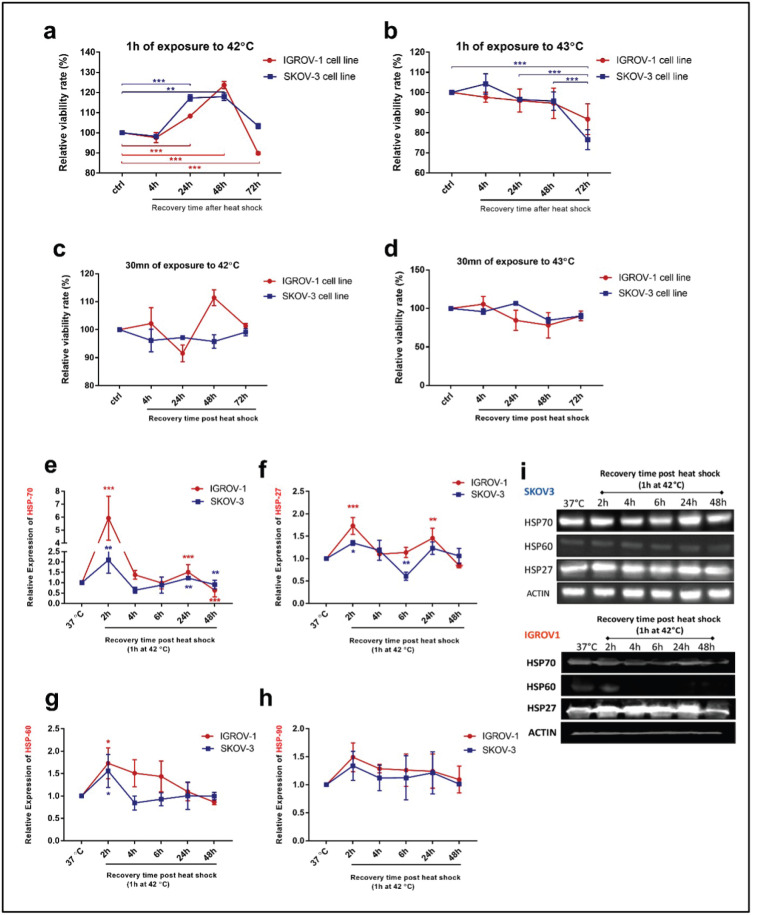
Cell viability and HSP expression pattern after hyperthermic treatment IGROV1 and SKOV3 cell lines were subjected to HT at (a and c): 42°C and (b and d): 43°C for different time courses (30 minutes and 1 hour). Cell viability is expressed as a percentage of control non-heat-treated cells (37°C) at 4, 24, 48, and 72 hours post treatment. Data shown are mean values (*n* = 5) ± SD and represent one of three independent experiments. *p < 0.05, **p < 0.01, ***p < 0.001 as indicated. Relative gene expression patterns of (e): HSP70, (f): HSP27, (g): HSP60 and (h): HSP90 were measured at 2, 4, 6, 24 and 48 hours post exposure to 42°C for 1 hour. Data were normalised to β-actin and expressed as mean ± SD of triplicates. *p < 0.05, **p < 0.01, ***p < 0.001 as indicated. (i): Western blot analysis of cell supernatants showing a continuous induction of HSP27 and HSP70 protein expression at 24 hours post exposure to 42°C for 1 hour. β-actin was used as a loading control (bands were cropped from original images).

Viability assays were performed to determine the optimal conditions of hyperthermic exposure on SKOV3 and IGROV1 cell lines (Figure 1a–d). Since 42°C and 43°C are the two most frequently used temperatures in the clinical application of loco regional HT in ovarian cancer, we tested those temperatures for two time courses (30 minutes and 1 hour). Cell viability was assayed at 4, 24, 48 and 72 hours post hyperthermic exposure. Data showed that exposing cells to either 42°C or 43°C over short time course (30 minutes), did not induce a significant effect on the viability levels in both cell lines (Figure 1c and d). However, when cells were exposed for 1 hour to 43°C, there was a significant decline in viability levels for both cell lines throughout the whole recovery period after heat shock (Figure 1b). On the other hand, exposure to 42°C for 1 hour induced a slight decrease of the viability levels at 4 hours post heat shock followed by a gradual increase at 24 and 48 hours compared to control (Figure 1a). This recovery might be due to the induction and synthesis of HSP. In fact, time monitoring of HSP expression showed that both HSP27 and HSP70 RNA and protein levels increased remarkably by 2 hours post exposure, and, interestingly, continued to be induced at 24 hours post heat shock with peak normalised values of 5.9 and 2.1 times the control for Hsp70 mRNA levels (Figure 1e) and of 1.73 and 1.35 times the control for HSP27 mRNA levels (Figure 1f) in IGROV1 and SKOV3 cells respectively. HSP60 significantly enhanced mRNA levels were noticeable only at 2 hours post heat shock (Figure 1g) while HSP90 was less responsive to heat exposure (Figure 1h). A similar expression pattern was observed at the protein levels (Figures 1i and S1a).

### Heat shock downregulated PD-L1 and NLRC5 expression in coculture system but promoted proinflammatory cytokines expression in PBMC

**Figure 2.**
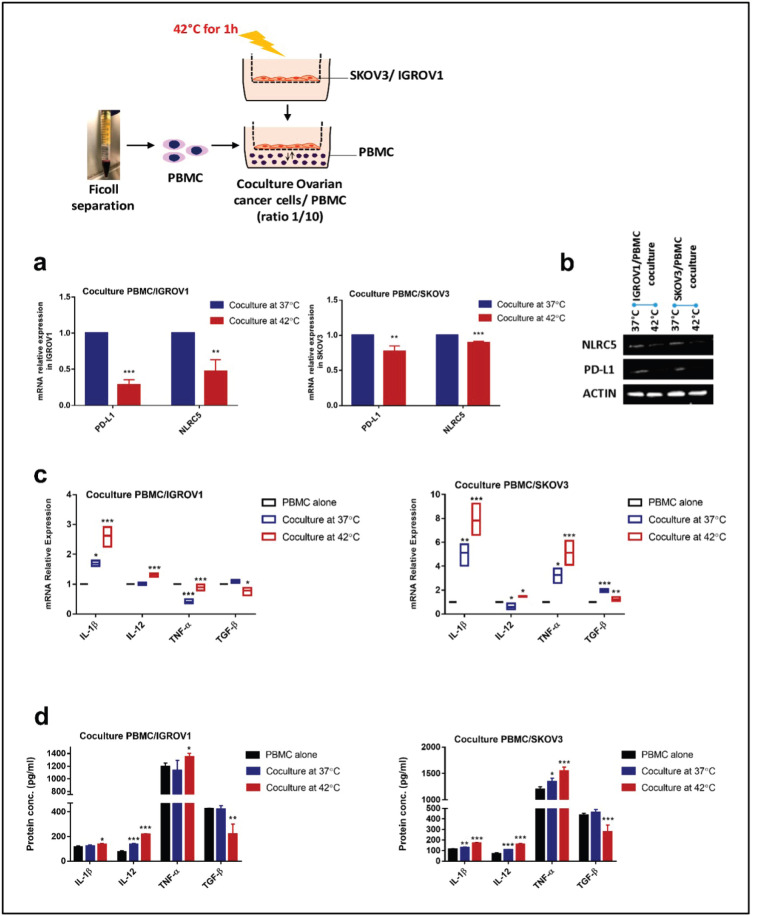
HT reduced the expression of PD-L1 and NLRC5 in coculture but promoted proinflammatory cytokines expression. In coculture conditions, PD-L1 and NLRC5 mRNA expression levels (a): decreased significantly post hyperthermic exposure. Data are the mean ± SD from three independent experiments with differences calculated using the delta-delta Ct method relative to the expression of reference gene β-actin; with *p-* values represented as follows: * < 0.05, ** < 0.01, *** < 0.001. (b): Representative Western blot showing the change in protein levels of PD-L1 and NLRC5 with β-actin used as a loading control. Three independent experiments were carried out and a representative image is shown (bands were cropped from original images). (c): Relative mRNA expression of IL-1ẞ, IL-12, TNF-α, and TGF-β1 in PBMC, cocultured with IGROV1 or SKOV3 exposed to HT. Expression was measured with qPCR and normalised to that of β-actin. (d): IL-1ẞ, IL-12, TNF-α, and TGF-β1 secretion levels in the supernatants measured by sandwich ELISA. Data are expressed as the mean ± SD of triplicates. *p < 0.05, **p < 0.01, ***p < 0.001, as indicated.

We next established a coculture system with PBMC to provide a more representative model of the *in vivo* situation, then we assessed the effect of heat on PD-L1 and NLRC5 expression. Results indicated that exposure to 42°C for 1 hour induced a significant concomitant reduction of PD-L1 and NLRC5 expression in ovarian cancer cell lines cocultured with PBMC at both mRNA (PD-L1: 0.2824 ± 0.041-fold change, 0.77 ± 0.044-fold change, NLRC5: 0.46 ± 0.08-fold change, 0.8967 ± 0.01098 in IGROV1 and SKOV3 respectively) as well as the protein levels (Figure 2a and b). To measure the inflammatory response to HT, IL-1β, TNF-α, IL-12 and TGF-β expression levels were determined by qPCR and ELISA. Results showed that pro-inflammatory cytokines IL-1β, TNF-α and IL-12 mRNA and protein expression levels increased substantially in heat-treated coculture systems when compared to non-heat-treated ones (Figure 2c and d). Conversely, the immunosuppressive cytokine TGF-β levels decreased significantly when heat was applied (Figure 2c and d).

### Conditioned medium from heat shocked ovarian cancer cells upregulated PD-L1 and NLRC5 expression in a positively correlated manner along with STAT3

**Figure 3.**
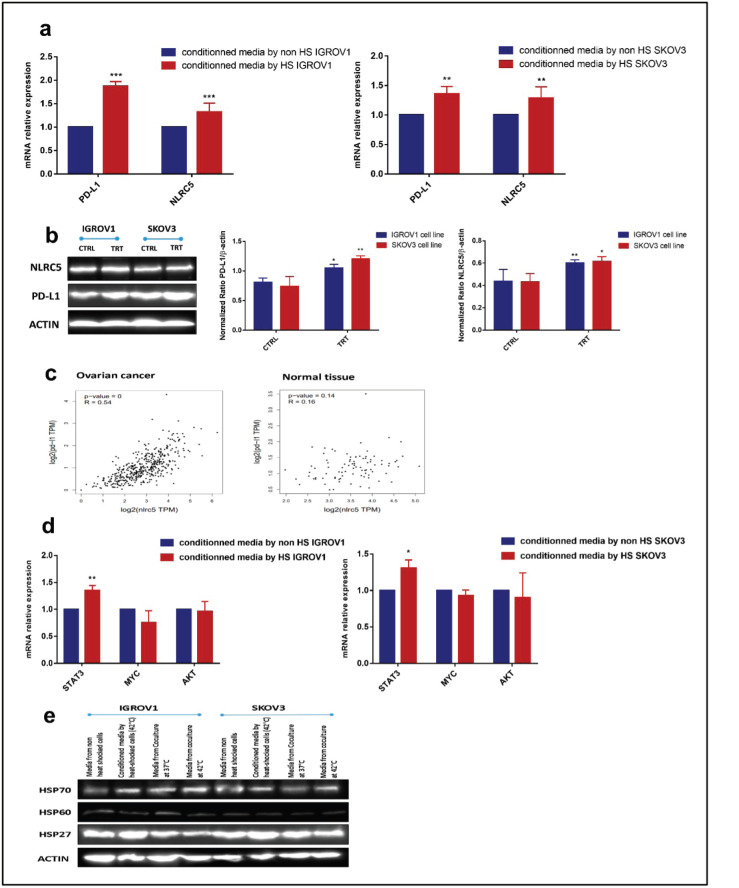
Conditioned media by heat-shocked ovarian cancer cells upregulated PD-L1 and NLRC5 in a positively correlated manner. Conditioned media by heat-shocked IGROV1 or SKOV3 significantly enhanced PD-L1 and NLRC5 expression at (a): mRNA and (b): protein levels compared to the conditioned media by non-heat shocked IGROV1 or SKOV3 (**p < 0.01, ***p < 0.001). (c): Relationship between PD-L1 and NLRC5 levels in ovarian cancer and normal tissue was evaluated using the TCGA dataset. A scatter plot of data points showed that PD-L1 expression is positively correlated with NLRC5 expression (Pearson’s *r* = 0.54, *p* < 0.001), while no significant correlation was found in normal tissue. (d): The effect of conditioned media by heat-shocked ovarian cancer cell lines on the mRNA expression levels of three PD-L1 transcriptional key regulators (STAT3, AKT, MYC) was evaluated by qPCR and normalised to that of β-actin. Data are expressed as the mean ± SD of triplicates. *p < 0.05, **p < 0.01, ***p < 0.001, as indicated. (e): Representative Western blot showing protein levels of HSP27, HSP60, and HSP70 in media from non-heat-treated cells, heat-treated cells, non-heated coculture systems, and heat-treated coculture systems. β-actin was used as a loading control.

As the expression of both PD-L1 and NLRC5 showed inhibition by HT in coculture conditions, we next sought to investigate the mechanisms involved in this downregulation. In order to determine whether the secreted soluble factors in response to heat contributed to the effect on PD-L1 and NLRC5 expression, conditioned culture medium by heat shocked IGROV1 and SKOV3 cells was used. Interestingly, the medium conditioned by heat shocked cancer cells induced the opposite effect with a significant concomitant increase in both PD-L1 and NLRC5 expression compared to previous coculture conditions as seen in Figure 3a and b. To explain this co-variation, the bioinformatics tool GEPIA was used to analyse the correlation between PD-L1 and NLRC5 expression using TCGA ovarian cancer dataset. As shown in Figure 3c, PD-L1 and NLRC5 are positively correlated (Pearson’s *r* = 0.54, *p*-value < 0.001) in ovarian cancer but not in normal tissue. The fact that NLRC5 gene expression decreased with the decrease in PD-L1 expression in the coculture system and vice versa in the conditioned medium experiments, led us to assume that these genes are controlled by the same regulatory networks and are functionally related. The regulatory mechanisms underlying PD-L1 expression are well documented and are either triggered by intrinsic oncogenic stimuli mainly MYC, STAT3 and AKT or extrinsic stimuli [31]. The understanding of mechanisms controlling NLRC5 expression is quite limited. Therefore, STAT3, MYC, and AKT mRNA expressions were evaluated using qPCR with results revealing that STAT3 expression was significantly upregulated in IGROV1 (1.36 ± 0.05 -fold) (*p* ˂ 0.005) and SKOV3 (1.31 ± 0.06 -fold) (*p* ˂ 0.05) cells treated with conditioned medium compared with control (Figure 3d). Comparative analysis of secreted HSPs levels in media conditioned by heat-shocked cells and media collected from coculture system was also conducted by Western blot and revealed higher expression of HSP27 in conditioned media compared to heated coculture systems (Figures 3e and S1b). No significant changes were observed in HSP70 levels between the two experimental conditions (monoculture versus coculture with PBMC) (Figures 3e and S1b).

### Individual HSPB1 knockdown and STAT3 phosphorylation inhibition downregulated partially PD-L1 and NLRC5 expression while combined inhibition downregulated completely PD-L1 and NLRC5 expression

**Figure S2.**
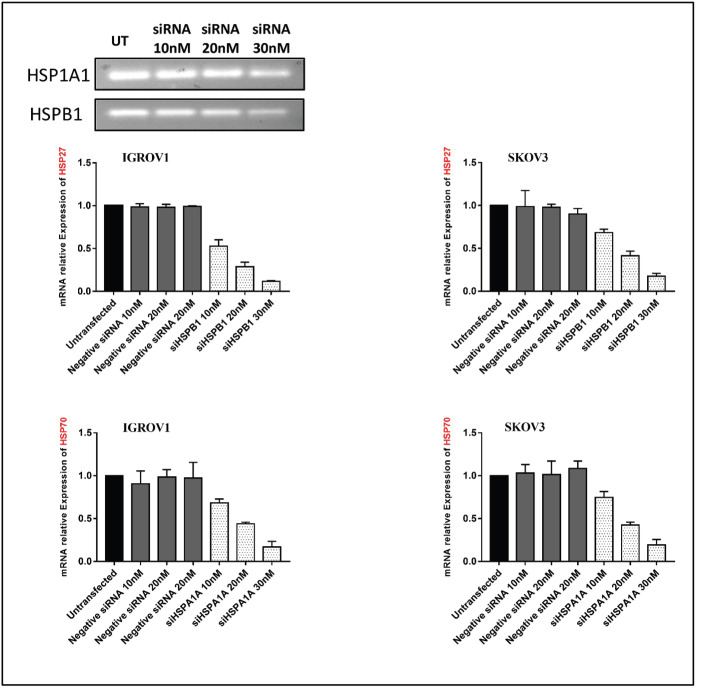
qPCR analysis of HSP27 and HSP70 gene expression following siRNA transfection in ovarian cancer cell lines IGROV1 and SKOV3. β-actin was used as an internal control.

**Figure S3.**
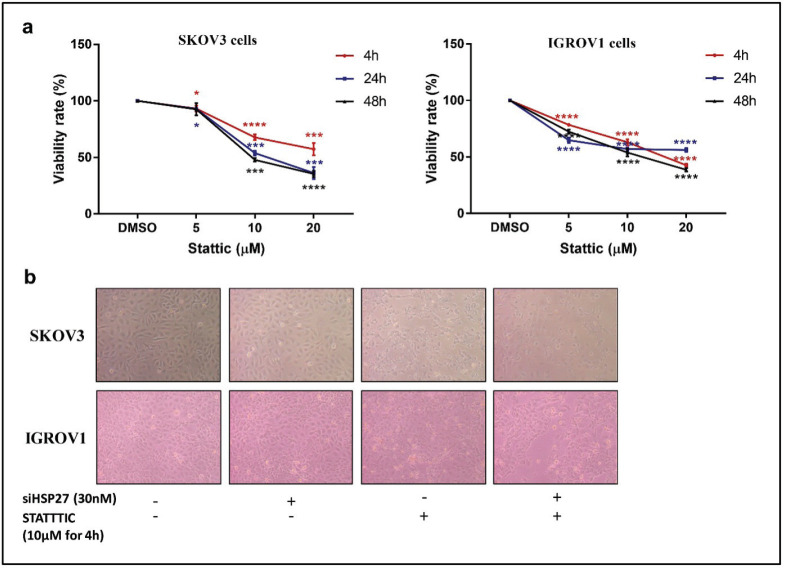
STATTIC effect on cell proliferation. (a): Relative cell viability of IGROV1 and SKOV3 cells treated with increasing doses of STATTIC (5, 10, 20 µM) at 4, 24 or 48 hours. Data are expressed as the mean ± SD of triplicates. *p < 0.05, **p < 0.01, ***p < 0.001, as indicated. (b): Representative photomicrographs showing the proliferation of IGROV1 and SKOV3 cells upon HSPB1 knockdown with siRNA (30 nM), STATTIC treatment (10 µM for 4 hours) and combined treatment.

**Figure 4.**
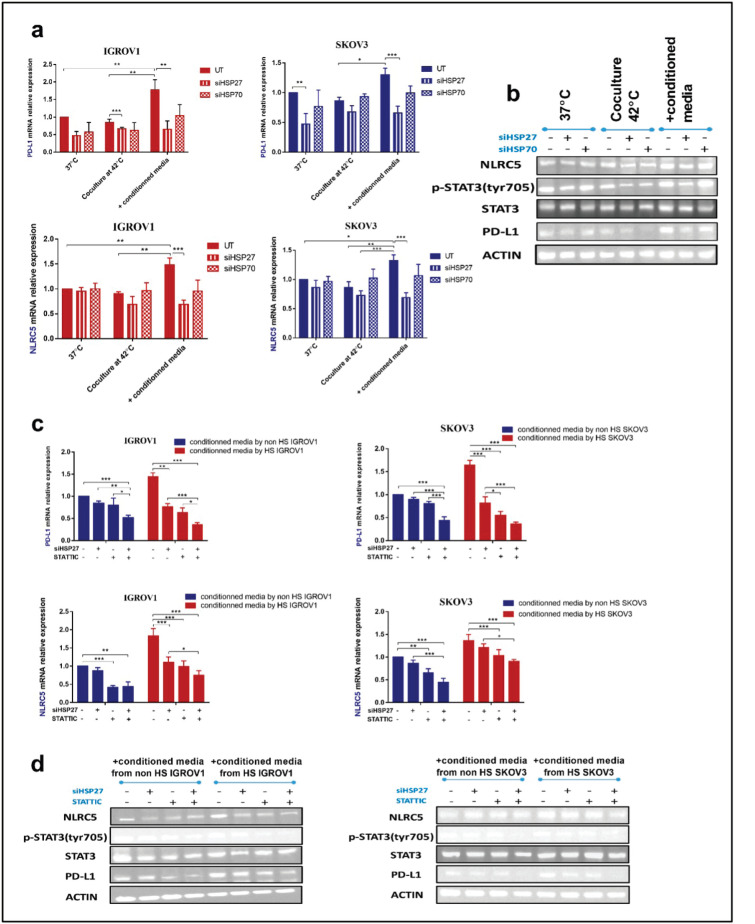
Combined inhibition of HSPB1 and STAT3 phosphorylation exerts a strong synergistic downregulation of PD-L1 and NLRC5. Knockdown of HSP27 and HSP70 was confirmed by qPCR and agarose gel electrophoresis (Figure S2). Significant downregulation of both PD-L1 and NLRC5 following HSPB1 silencing at (a): both mRNA and (b): protein levels. No significant variation was noted with HSPA1A knockdown. (b): SiHSP27 also attenuated phospho-STAT3 levels. (c): Relative mRNA expression levels of PD-L1 and NLRC5 showing an overall significant decrease after STATTIC treatment (10 µM for 4 hours). Combined treatment with STATTIC and siHSP27 appears to induce a more pronounced significant decrease. Expression was measured with qPCR and normalised to that of β-actin. The data are representative of at least three independent experiments and are expressed as the mean ± SD. *p < 0.05, **p < 0.01, ***p < 0.001, as indicated. (d): Western blot analysis demonstrated a similar effect. β-actin was used as a loading control (bands were cropped to improve clarity).

Based on the above results indicating the upregulation of both PD-L1 and NLRC5 upon adding the conditioned medium by heat-shocked cells, gene silencing of HSP27 and HSP70 was induced by siRNA. Those two HSPs were inhibited since their expression and secretion have been shown to remain high at 24 hours post hyperthermic exposure. qRT-PCR and agarose gel electrophoresis performed following siRNA transfection confirmed HSPA1A and HSPB1 downregulation (Figure S2). Results revealed a significant downregulation of both PD-L1 and NLRC5 following the silencing of HSP27 (siHSPB1 30 nM) at both mRNA and protein levels (Figure 4a and b). However, HSP70 knockdown had no significant effect on PD-L1 and NLRC5 levels. Moreover, the activation of STAT3 was blocked using the pharmacological inhibitor STATTIC at the chosen concentration of 10 µM for 4 hours. This concentration and incubation duration were the most favourable conditions with respect to maintenance of cell viability (67.74% for SKOV3 and 63.02% for IGROV1 cells) and, thus, the most suitable for determining the effect on gene expression (Figure S3). We have found that individual inhibition of HSP27 and STAT3 resulted in partial downregulation of PDL1 and NLRC5 protein and gene expression, while the combined HSP27 and STAT3 resulted in a more pronounced downregulation of PD-L1 and NLRC5 expression (Figure 4c and d).

## Discussion

In the past two decades, the emergence of immunotherapy has revolutionised the treatment of multiple solid tumour types. Though modest, the presence of responses to immunotherapy in heavily pretreated patients with advanced ovarian cancer is promising and has prompted further investigation concerning means that lead to advanced immunotherapy and overcoming resistance [32].

HT has been shown to increase the overall survival of patients with advanced ovarian cancer when combined with chemotherapy in multiple clinical trials [7]. Moreover, extensive preclinical data clearly show the potential of HT to improve immunotherapy outcomes [33]. Indeed, the HSPs expressed upon heat shock were reported to enhance immune cell immunogenicity by activating macrophages function, inducing DC maturation, and increasing NK and cytotoxic T cells cytotoxicity [10]. However, little is known about the effects of HT on immune evasion mechanisms responsible for limiting the efficacy of immunotherapy.

In that context, we started our study by performing a viability assay in two ovarian cancer cell lines (IGROV1 and SKOV3) to determine the cellular response to heat stress (42°C and 43°C for 30 or 60 minutes). Our results showed a reduction in viability levels at 43°C, which can be explained by the capacity of HT to induce cell cycle arrest. A more pronounced reduction was observed in response to 60 minutes exposure compared to 30 minutes. Indeed, Skitzki *et al* [22] confirmed that significant cell killing could be achieved by heating cells and tissues to temperatures above 42°C for 1 or more hours, and Furusawa [34] demonstrated that heat-induced cell cycle responses depend primarily on the duration of the heating at a given temperature. In contrast, when cells were exposed to 42°C for 60 minutes, there was a slight decrease in viability rates at 4 hours post exposure, followed by a gradual recovery with an increase in viability rates compared to control cells at both 24 and 48 hours. This increase might be explained by the rapid cell proliferation following recovery from heat damages due to the induction of HSPs expression. In fact, in addition to their cytoprotective functions, HSPs have anti-apoptotic properties and seem to be able to block the cell death process at different levels, mainly via HSP27 and HSP70 [35]. In this regard, Lanneau *et al* [36] reported that HSPs play a critical role in maintaining the balance between cell death and survival through modulating several signalling cascades such as JNK, AKT, and NF-*κ*B. The ability of cells to recover following heat shock and gain thermotolerance explains why several studies indicate that HT modalities, when applied alone, are insufficient to kill cancer cells. Still, they can improve the cytotoxic effects of chemotherapy and radiation. Hildebrandt *et al* [37] confirmed earlier that malignant cells exposed to temperatures ˂43°C have impaired susceptibility to heat-induced cytotoxicity and can develop thermotolerance due to the induction of HSPs. We, therefore, analysed the kinetics of HSPs expression post heat shock (42°C for 60 minutes). Our results showed that by 2 hours post thermal stress, HSP27, HSP70, and HSP60 expression increased significantly and reached their maximum levels. By 24 hours post thermal stress, only HSP27, HSP70 mRNA, and protein levels continued to increase significantly compared with control (cells maintained at 37°C). A similar pattern of thermally induced HSPs expression by mesenchymal stem cells was described by Moloney *et al* [38]. This transient upregulation of the anti-apoptotic HSP27 and HSP70 expression up to 24 hours post heat shock may be sufficient to preserve cell viability and allow their survival following mild hyperthermic exposure.

The 60 minutes treatment at 42°C was selected for the rest of the experiments since this temperature and duration are less likely to damage cells in the tumour microenvironment but are still capable of inducing cellular stress response as demonstrated by the time-related expression profile of HSPs at the protein and mRNA levels.

To gain insight into the *in vivo* situation in response to heat, we established a coculture model of ovarian cancer cell lines and PBMC. We then measured the pro-inflammatory cytokines response when PBMC were cultured alone, in the presence of non-heated ovarian cancer cells or when these latter were exposed to HT. Results showed that IL-1β, IL-12, and TNF-α expression levels by PBMC increased significantly 24 hours post treatment in coculture exposed to 42°C. This increase can help overcome the immunosuppression within the tumour microenvironment. Indeed, several studies suggested that extracellular HSPs (HSP70, HSP90) might be potent boosters of the innate immunity system, capable of inducing the production of pro-inflammatory cytokines [39, 40]. Moreover, TGF-beta levels decreased significantly in coculture exposed to 42°C; this decrease could be beneficial since TGF-β has been known to favour the establishment of an immunosuppressive tumour microenvironment and has been associated with resistance to immunotherapeutic drugs [41, 42].

**Figure 5.**
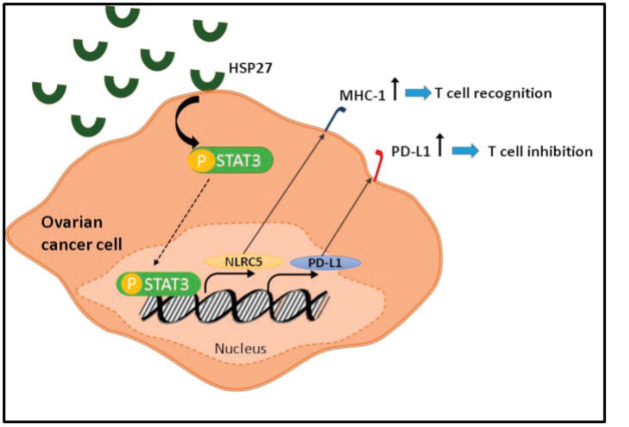
Schematic diagram showing the role of HSP27 in the regulation of both PD-L1 and NLRC5 via STAT3 activation.

Among the most critical mechanisms exploited by tumours to evade immune recognition is increasing the expression of inhibitory molecules such as PD-L1 and downregulating the expression of genes coding MHC class I molecules. With NLRC5 being the master selective transactivator of MHC class 1 genes, we sought to analyse its expression along with that of PD-L1 in the coculture system [27]. Our results revealed a concomitant decrease of both PD-L1 and NLRC5 in IGROV1 and SKOV3 cells cocultured with PBMC at 24 hours post heat shock. To further understand this response and determine whether the extracellular HSPs expressed upon heat shock were implicated, a conditioned medium from heat-treated ovarian cancer cells extracted at 24 hours post exposure was applied to non-heated ovarian cancer cells. Then, we measured PD-L1 and NLRC5 levels. Conversely to coculture conditions, the use of conditioned media induced the opposite effect with a significant concomitant increase of both PD-L1 and NLRC5 expression. This co-variation in the coculture and monoculture settings led us to analyse the correlation between these two genes using the GEPIA tool. Results indicated a significant positive correlation between NLRC5 and PD-L1 in ovarian cancer. Since NLRC5’s primary function is to selectively activate MHC class 1 genes and other genes involved in the MHC class 1 antigen presentation pathway, the positive correlation between PD-L1 and NLRC5 suggests that PD-L1 upregulation and MHC class 1 downregulation are two mutually exclusive mechanisms of immune evasion used by ovarian cancer cells; meaning that when one of them is deployed, the other is repressed. Therefore, recovering MHC class 1 genes via NLRC5 targeting may represent a promising strategy to increase the efficacy of PD-1/PD-L1 inhibitors in ovarian cancer. This co-variation also led us to assume the presence of a common regulator of both PD-L1 and NLRC5 expression. As the latter increased when conditioned media from heat-shocked ovarian cancer cells was added to non-treated cells, and since our previous study of HSPs kinetics showed that mainly HSP27 and HSP70 accumulate in the media during the first 24 hours post heat shock, HSPB1 and HSPA1A genes were knocked down in cells from which conditioned media is collected. In parallel, AKT, MYC, and STAT3 key regulators of PD-L1 expression were analysed at the mRNA levels. Our results revealed that HSP27 knockdown inversed the increase of both PD-L1 and NLRC5 and induced a decrease in STAT3 phosphorylation and mRNA levels. In fact, HSP27 is involved in regulating a significant number of cellular processes such as signal transduction, transcription, and translation mechanisms and has a wide range of client proteins [43]. By collecting data from the existing scientific literature, Arrigo [44] have built a comprehensive interactome that lists the proteins known to interact with HSP27. STAT3 was found among cancer cells HSP27 client proteins. In a similar manner to our results, Shochet *et al* [45] found that HSP27 silencing reduced STAT3 levels and phosphorylation in extravillous trophoblast during placental implantation. Furthermore, Rocchi *et al* [46] described the promotion of the STAT3 signalling pathway by HSP27. They have also revealed a direct interaction between HSP27 and STAT3 and that STAT3 levels correlated with HSP27 levels in prostate cancer. Interestingly, a recently published review has discussed the therapeutic potential of targeting HSPs along with the PD-1/PD-L1 immune checkpoint axis in patients with myeloproliferative neoplasms without establishing the link or the interaction between these two targets [47]. On the other hand, considerable evidence supports the regulation of PD-L1 by STAT3 [48]. Prestipino *et al* [49] showed that phosphorylated STAT3 stimulates PD-L1 promoter activity and protein expression. In T cell lymphoma, Marzec *et al* [50] found that STAT3 upregulates PD-L1 expression by binding to the PD-L1 promoter; this effect can be suppressed by silencing STAT3 with siRNA. Additionally, Li *et al* [51] demonstrated that inhibiting JAK2/STAT3 pathway inhibited NLRC5 expression. Based on the above findings, we investigated whether PD-L1 and NLRC5 decrease following HSP27 silencing might be linked to STAT3 activity. STATTIC, a selective inhibitor of STAT3 activation, dimerisation, and nuclear translocation, was added to cells prior to treatment with conditioned media from heat-shocked cells. A dual treatment with STAT3 and HSPB1 siRNA was also performed. Our results showed that individual inhibition of HSP27 and STAT3 has partially downregulated PDL1 and NLRC5 protein and gene expression, while combined HSP27 and STAT3 inhibition has completely downregulated PD-L1 and NLRC5 expression. This result suggests that HSP27 regulates PD-L1 and NLRC5 expression by modulating STAT3 phosphorylation (Figure 5).

As for the decrease in PD-L1 and NLRC5 expression observed in cocultured conditions, it might be explained by the considerable increase in pro-inflammatory cytokines expression post heat treatment. In fact, Nahomi *et al* [52] have demonstrated that pro-inflammatory cytokines significantly downregulated HSP27 levels by inducing the formation of reactive oxygen species and nitric oxide in human retinal capillary endothelial cells. This is in line with our western blot analysis results that showed a significant decrease in HSP27 levels in media collected from heated ovarian cancer cells / PBMC coculture (1 hour at 42°C) compared to heated ovarian cancer cells monoculture (1 hour at 42°C).

## Conclusion

We demonstrated that PD-L1 and NLRC5 expressions are positively correlated in ovarian cancer and that they co-vary in tandem in response to heat stress. Therefore, PD-L1 upregulation and MHC class 1 downregulation can be considered as two mutually exclusive mechanisms of tumoural immune evasion. This finding highlights the therapeutic potential of combined targeting of the PD-1/PD-L1 axis and NLRC5 to recover MHC class 1 and thus increase the efficacy of immune checkpoint inhibitors. We have also found out that, by modulating STAT3 levels and phosphorylation, HSP27 regulates both PD-L1 and NLRC5. Further investigation is required to probe the extent of involvement of STAT3 in PD-L1 and NLRC5 upregulation.

## List of abbreviations

DC, dendritic cells; HIPEC, hyperthermic intraperitoneal chemotherapy; HSPs, heat shock proteins; HT, hyperthermia; MICA, MHC class I polypeptide related sequence A; NK, natural killer; NLRC5, NOD-like receptor (NLR) family, caspase recruitment (CARD) domain containing 5; PBMC, peripheral blood mononuclear cells; PD-1, programmed death-1; PD-L1, programmed death-ligand 1; TCGA, the cancer genome atlas; TCR, T-cell receptor; TILs, tumour-infiltrating T-cells.

## Ethics approval and consent to participate

This study was approved by the Ethics committee of the Saint Joseph University of Beirut and was conducted in compliance with the Declaration of Helsinki (as revised in 2013). The participants were informed and provided written informed consent for the scientific research use of blood samples.

## Consent for publication

Not applicable.

## Availability of data and materials

All data generated and analysed during this study are available from the corresponding author on reasonable request.

## Conflicts of interest

The authors declare that they have no competing interests.

## Funding

This study was funded by the Research Council of the Saint Joseph University of Beirut.

## Authors’ contributions

MF carried out the experiments, performed the analysis, and wrote the manuscript with support from GH. GH proposed the conditioned medium experiments. RT contributed to the interpretation of the results. MM revised the manuscript. DA conceived the original idea and supervised the project. All authors provided critical feedback and helped shape the analysis and the manuscript.

## Figures and Tables

**Table S1.** List of primers used for this study.

Primers	Forward	Reverse
HSPB1	5′- CCAACGAGATCACCATCCCA-3′	5′- TTACTTGGCGGCAGTCTCAT-3′
HSPA1	5′- CTACTTCAACGACTCGCAGC -3′	5′- CCAGGTCAAAGATGAGCACG -3′
HSP60	5′- TGGAAGAAAGGGTGTCATCA -3′	5′- TCACTCAACAGAACATAGGCATC -3′
HSP90	5′- TGGGAAGATCACTTGGCAGT -3′	5′- GCACGTCGTGGGACAAATAG -3′
PDL1(CD274)	5′-TGGATCCAGTCACCTCTGAAC-3′	5′-AGGACTTGATGGTCACTGCT-3′
NLRC5	5′-CATCTCGGACCTCTTCAACA-3′	5′-GGCCTGGAAACAGTTCAGAT-3′
IL-1ẞ	5′-CAGAAGTACCTGAGCTCGCC-3′	5′-AGATTCGTAGCTGGATGCCGA-3′
IL-12	5′-CCAGAAGGCCAGACAAACTC-3′	5′-CTCTGGAATTTAGGCAACTCTCA-3′
TGF-ẞ	5′-CGCGTGCTAATGGTGGAAA-3′	5′-GTGTGTACTCTGCTTGAACTTTCA-3′
TNF-α	5′-GGTATGAGCCCATCTATCTG-3′	5′-TTTTTGAGCCAGAAGAGGTT-3′
AKT	5′-CAAAGAAGTCAAAGGGGCTGC-3′	5′-CAGCCAACCCTCCTTCACAA-3′
c-Myc	5′-CACCACCAGCAGCGACTCT-3′	5′-TTCCACAGAAACAACATCGATTTC-3′
STAT3	5′-CTGCCCCATACCTGAAGACC-3′	5′-GGGTTCAGCACCTTCACCAT-3′
ACTB	5′-CTGTGCTATCCCTGTACGCC-3′	5′- GAGGGCATACCCCTCGTAGA-3′
